# The Ethanolic Extract of *Piper glabratum* Kunth Is Teratogenic and Interferes with the Ossification Process of Swiss Mice Fetuses

**DOI:** 10.3390/ph16030430

**Published:** 2023-03-11

**Authors:** Rogério Carlos Sanfelice Nunes, Silvia Cordeiro das Neves, Fabricia Rodrigues Salustriano, Marcelo Luiz Brandão Vilela, Valter Aragão do Nascimento, Karuppusamy Arunachalam, Roberto da Silva Gomes, Candida Aparecida Leite Kassuya, Jonas da Silva Mota, Rodrigo Juliano Oliveira

**Affiliations:** 1 Centro de Estudos em Células-Tronco, Terapia Celular e Genética Toxicológica (CeTroGen), Faculdade de Medicina (FAMED), Universidade Federal de Mato Grosso do Sul (UFMS), Campo Grande 79070-900, Mato Grosso do Sul, Brazil; 2 Programa de Pós-graduação em Saúde e Desenvolvimento da Região Centro-Oeste, Faculdade de Medicina (FAMED), Universidade Federal de Mato Grosso do Sul (UFMS), Campo Grande 79070-900, Mato Grosso do Sul, Brazil; 3 Faculdade de Medicina Dr. Hélio Mandetta (FAMED), Universidade Federal de Mato Grosso do Sul (UFMS), Campo Grande 79070-900, Mato Grosso do Sul, Brazil; 4 Department of Pharmaceutical Sciences, North Dakota State University, Fargo, ND 58102, USA; 5 Faculdade de Ciências da Saúde, Universidade Federal da Grande Dourados (UFGD), Dourados 79825-070, Mato Grosso do Sul, Brazil; 6 Departamento de Química, Universidade Estadual de Mato Grosso do Sul (UEMS), Dourados 79804-970, Mato Grosso do Sul, Brazil

**Keywords:** Piperaceae, medicinal plant, malformation, bone, embryofetal development, *Piper glabratum* Kunth

## Abstract

*Piper glabratum* Kunth is a plant traditionally used to treat pain and inflammation in the Mato Grosso do Sul, Brazil. Even pregnant women consume this plant. Toxicology studies of the ethanolic extract from the leaves of *P. glabratum* (EEPg) could establish the safety of popular use of *P. glabratrum*. Thus, the effects of the ethanolic extract of leaves of *P. glabratum* (EEPg) on the reproductive performance and embryofetal development of Swiss mice were evaluated. Pregnant female mice were treated with 100, 1000 and 2000 mg/kg throughout the gestational period by gavage (p.o). The control group received the EEPg vehicle (Tween 80–1%) in the proportion of 0.1 mL/10 g (p.o.). The results demonstrated that EEPg has low maternal toxic potential and does not alter the reproductive performance of females. However, it altered embryofetal development and caused fetal weight reduction (increasing the frequency of small-for-gestational-age fetuses) at the two highest doses. In addition, it interfered with placental weight, placental index and placental efficiency. The frequency of visceral malformations increased by 2.8 times for the lowest dose of EEPg, and skeletal malformations increased by 2.48, 1.89 and 2.11 times for doses of 100, 1000 and 2000 mg/kg of EEPg, respectively. It is noteworthy that 100% of the offspring treated with EEPg showed changes in the ossification process. Thus, it is considered that the EEPg has low maternal toxic potential; it does not alter the reproductive performance of females. However, it is teratogenic and interferes, mainly, in the ossification process, and therefore its use is contraindicated in the gestational period.

## 1. Introduction

The genus Piper, belonging to the Piperaceae family, is popularly known as “pimenta”, “pariparoba caapeba” and “falso jaborandi” [1,2,3,4]. This genus has about 2000 species geographically distributed in tropical and subtropical regions [2,3,5]. Generally, these plants are shrubs with simple alternate leaves and segmented branches united by nodes [5]. One of the species of this genus is *Piper glabratum* Kunth. This species, which occurs in Mato Grosso do Sul, Brazil, is popularly used in that state to treat pain and inflammation [4].

A seller of homemade remedies and medicinal plants (traditional culture) from the Municipal Market of Campo Grande, MS, Brazil, considered the use of leaf decoction (tea) of *P. glabratum* for all ages and for pregnant women. For the method of preparation, specific quantities are not described (one tablespoon of powdered dry leaves (5 g) for one liter of water was mentioned), and the population collects this plant in the forest (or buys the dried leaves) to prepare the tea. The mode of use in folk medicine is to take a liter of tea throughout the day and night.

There have been relatively few studies on this species. However, Branquinho et al. (2017) [3] demonstrated the anti-inflammatory effects of the essential oil from the leaves of *P. glabratum* evaluated in Swiss mice, confirming the ethnopharmacological indication of this plant. Later, Leitão et al. [4] showed that the ethanolic extract of the leaves of this species, particularly the hexane fraction, has antihyperalgesic and anti-inflammatory properties.

For this species, the antiparasitic activity against Leishmania sp. and *Trypanosoma cruzi* are recorded [6]. The literature also records that methanolic extract from the roots of *P. glabratum* has a diuretic effect and can be hepatotoxic in Wistar rats when used in high doses intraperitoneal [2]. However, the LD_50_ orally is greater than 3000 mg/kg (for males and females) and intraperitoneally is 2426.216 mg/kg for females and greater than 3000 mg/kg for male animals [2].

The essential oils of *P. glabratum* leaves have no acute toxicity up to a dose of 5000 mg/kg and no subacute toxicity up to a dose of 1000 mg/kg in female Swiss mice [3]. The LD_50_ in *Wistar* rats is greater than 2000 mg/kg for the ethanolic extract of the leaves. However, the animals showed signs of central nervous system depression, such as drowsiness, in the first 6 h after treatment with the ethanolic extract [4].

The chromatographic fractionation of the hexane fraction demonstrated the presence of Phytol and a mixture of Stigmasterol and β-sitosterol. The hydroalcoholic fraction indicated the presence of glycosylated flavanols [4]. The crude extract studied by Leitão et al. [4], which has an antihyperalgesic and anti-inflammatory effect, was the same one tested in the present research.

As observed in the literature review, some important biological properties have been already demonstrated by the ethanolic extract such as being antihyperalgesic and anti-inflammatory (previously described by our group) [4]. Pregnant women are affected by inflammatory processes and pain. Therefore, they are candidates to be exposed to substances present in medicinal plants popularly indicated to treat pain and inflammation. Given the above, it is important to establish the safety of using the ethanolic extract obtained from *P. glabratum* during pregnancy. Given the above, the present research aimed to evaluate the effects of the ethanolic extract of *P. glabratum* on the reproductive performance and embryofetal development of Swiss mice.

## 2. Results

### 2.1. Biometric Parameters

Initial weight and net weight gain did not vary between experimental groups (*p* > 0.05). The final weight, weight gain and uterus weight increased (*p* < 0.05) in the group EEPg 100 mg/kg compared to the groups EEPg 1000 and 2000 mg/kg. However, EEPg 100 mg/kg and the control group did not show differences between them (*p* >0.05) (Table 1).

The absolute weight of the heart, lungs, liver, kidneys and spleen and the relative weight of the heart, kidneys and spleen did not differ (*p* > 0.05) between the experimental groups. However, the relative lung weight increased (*p* < 0.05) in the EEPg 1000 and 2000 mg/kg groups compared to the control and EEPg 100 mg/kg groups. The relative liver weight increased (*p* < 0.05) in the EEPg 2000 mg/kg group compared to the other experimental groups (Table 2).

### 2.2. Reproductive Performance

The number of pregnant female animals, implants, live and dead fetuses and resorption as well as fetal viability, rate of post-implantation losses, rate of resorption and sex ratio did not vary (*p* > 0.05) between different experimental groups (Table 3).

### 2.3. Embryo-Fetal Development

Fetal weight decreased (*p* < 0.05) in the EEPg 1000 and 2000 mg/kg groups, which consequently led to an increase (*p* < 0.05) in the number of fetuses classified as small for gestational age (SGA). Placental weight decreased (*p* < 0.05) in the EEPg 1000 mg/kg group compared to the other experimental groups. For this same group, a reduction (*p* < 0.05) of the placental index and an increase (*p* < 0.05) of the placental efficiency were observed. An increase in the placental index was also observed for the EEPg 2000 mg/kg in relation to the other groups (Table 4).

The external malformations observed in the different experimental groups were Hydropsy, kyphosis, hyperflexion and hyperextension of the unilateral anterior and posterior limb, hyperflexion and hyperextension of the bilateral posterior limb, small paws and a bent tail. The frequency of malformed fetuses and the percentage of malformations did not vary (*p* > 0.05) between the experimental groups, except for the frequency of kyphosis that occurred more (*p* < 0.05) in the EEPg 100 mg/kg group compared to the control group (Table 5).

Visceral malformations observed were mild, moderate and severe hydrocephaly, choanae hyperplasia, bifurcated tongue and enlarged ventricular chamber (heart). In the individual analysis of the malformations, an increase (*p* < 0.05) in the frequency of moderate hydrocephalus was observed in the EEPg 2000 mg/kg in relation to the control group. For all other malformations, no significant differences were observed (*p* > 0.05). However, there was a 2.8× increase (*p* < 0.05) in the global frequency of malformations in the EEPg 100 mg/kg compared to the Control group. The percentage of malformed fetuses was 18.87%, 45.16%, 16.42% and 33.33% for the Control, EEPg 100, 1000 and 2000 mg/kg groups, respectively (Table 6).

The skeletal malformations observed in the head were right interparietal and orbitotransphenoid agenesis and reduced ossification of the frontal, interparietal, maxilla, nasal, parietal, premaxilla, supraoccipital and zygomatic bones. In the trunk, agenesis of the sternebral center and xiphoid process, arthrogryposis, misshapen sternebra, bifurcated rib, hyperkyphosis, hyperkyphosis with anteriorization of the head, reduced ossification of the rib, sternebra and xiphoid process and torsion of the vertebral centers were observed. In the limbs, agenesis of the phalanges, of the metacarpal and of the caudal arch and alteration of the nucleus of ossification of the knee and elbow were observed (Table 7).

There was an increase (*p* < 0.05) in the frequency of reduced ossification of the frontal, interparietal, nasal, parietal and supraoccipital regions in the groups treated with EEPg compared to the control group. There was also an increase (*p* < 0.05) in the frequency of the misshapen sternebra for these same groups in relation to the Control (Table 7).

There was also an increase (*p* < 0.05) in the frequency of reduced sternebral ossification and phalanx agenesis for the EEPg 1000 and 2000 mg/kg groups compared to the control group. The increase (*p* < 0.05) of agenesis of the sternebral center was observed only for the EEPg 1000 mg/kg group in relation to the Control group (Table 7).

A global analysis of the frequencies of skeletal malformations showed an increase (*p* < 0.05) of 2.48, 1.89 and 2.11 times for the EEPg at doses of 100, 1000 and 2000 mg/kg in relation to the control group, respectively. The percentage of malformed fetuses was 47.37% for the control group and 100% for the groups treated with EEPg (Table 7).

## 3. Discussion

The use of medicinal plants is an important strategy for providing Primary Health Care, and, in line with the World Health Organization and the United Nations Children’s Fund (UNICEF), the Unified Health System has developed public policies and national regulations regarding the use of traditional remedies with proven efficacy and the possible incorporation of this knowledge into primary health care activities [8,9].

Currently, the National List of Medicinal Plants of Interest for the SUS has about 70 species that are under analysis [10]. However, Brazilian populations, especially those far from large centers and who have difficulty accessing treatments, make continuous use of other species [11]. *P. glabratum* is not on the Renisus list. However, this plant is used to treat pain and inflammation according to traditional medicine in Mato Grosso do Sul [4] by different populations, including pregnant women.

The ethnopharmacological indication of this plant has already been proven [3,4]. However, there is no safety record of its use during pregnancy. This fact demonstrates the originality, the pioneers and the need for this study. We chose to test the EEPg described by Leitão et al. [4] and not the tea conventionally prepared by decoction by the population. The ethanolic extract can facilitate the bioprospecting of a standardized extract because this solvent is relatively safe for human consumption [12,13]. The standardized extracts have greater stability to be stored and consumed besides concentrating the active principles. Stability (chemical, physical, microbiological, therapeutic and toxicological) is the period in which a product retains, even when stored, the properties and characteristics it had at the time of its production [14]. This strategy is important for populations that do not have easy access to allopathic medicines [15].

Our data indicate that EEPg has low maternal toxic potential since it did not significantly alter biometric parameters and the relative and absolute weight of the organs. A slight reduction in the final weight, weight gain and uterine weight were observed in animals treated with the lowest dose of EEPg (100 mg/kg). A reduction in the absolute weight of the spleen was also observed. However, this difference was not maintained in the evaluation of relative weight. Thus, it is inferred that this difference is not due to treatment but is instead due to the size of the animals. An increase in the weight of the lungs was also observed for the two highest EEPg doses (1000 and 2000 mg/kg) and of the liver for the highest dose (2000 mg/kg). These results suggest attention.

Despite using another species, Leitão et al. [4] reported that at 2000 mg/kg in *Wistar* rats, it did not cause death or signs of toxicity in the acute toxicity test. It is also noteworthy that these animals did not show changes in the observed Hippocratic parameters, such as piloerection, tearing, changes in mucous membranes, eyes or feces or induced motor response such as tremors, irritability, contortion, convulsions and changes in breathing. In the macroscopic analysis of organs, no change (color, size and texture) was observed in relation to the control group. In our study, we performed the same evaluations and did not find changes over the eighteen consecutive days of treatment (corresponding to the gestational period). We point out that the extract used by Leitão et al. [4] and the one used in this study is the same. Furthermore, we highlight that these previously reported signs are important indicators of toxicity [16].

The EEPg did not alter the reproductive performance since no alteration was observed in the number of pregnant females, of implantations, of live and dead fetuses and of resorptions nor in the rates of post-implantation losses, resorption rate, fetal viability and sex ratio. These parameters are those considered by the literature in the area to predict the possible deleterious effect of extracts and/or compounds on the performance of females [17,18,19,20,21,22]. However, EEPg altered embryofetal development.

The EEPg reduced fetal weight for the two highest doses (1000 and 2000 mg/kg). Fetal weight reduction at a dose of 1000 mg/kg is associated with reduced placental weight, reduced placental index and increased placental efficiency. Reduction in fetal weight at a dose of 2000 mg/kg is associated with increased placental index without interfering with placental weight and placental efficiency. These facts resulted in an increase in fetuses classified as small for gestational age according to Soulimane-Mokhtari et al. [23]. This indicates an important effect of this extract on embryo-fetal development. No similar results were found in the consulted literature. However, in general, fetuses reduce their growth and/or stop gaining weight when there is a reduction in placental efficiency. However, the literature indicates different correlations between fetal weight, adequacy of weight for gestational age, placental weight, placental index and placental efficiency [17,18,19,21,22,24,25,26,27,28,29,30].

Placental efficiency refers to the ratio of fetal weight to placental weight in grams. This relationship establishes the weight of the fetus produced by the weight of the placenta that worked on this production of body mass [31,32,33,34]. This calculation estimates the maternal–fetal relationship established during the gestational period and is a determinant of intrauterine growth, since the placenta and its performance are responsible for the nutritional and hormonal supply that determines fetal growth and weight gain [29,30,31]. It is also known that the supply capacity of the placenta is directly influenced by its size, morphology, blood flow and transport efficiency [32,33,34,35,36]. In this context, fetuses that are growing less and that are generally associated with lower placental weights tend to trigger increased placental efficiency as a compensatory mechanism.

At the intermediate dose (EEPg 1000 mg/kg) there was a reduction in fetal weight associated with a reduction in placental weight and placental index and an increase in placental efficiency. This fact corroborates what was previously described. Thus, the outcome of these changes can lead to a reduction in fetal weight [34] which can determine an inappropriate weight for gestational age [23,37]. This fact was observed for this experimental group, as small-for-gestational-age fetuses increased by 9.87 times. The same fact was not observed for the highest dose (EEPg 2000 mg/kg); that is, for this group, there was a reduction in fetal weight and a 7.35 times increase in the number of fetuses small for gestational age in relation to the control group. However, for this group, there was no change in placental weight. However, an increase in the placental index was observed without alteration of placental efficiency.

EEPg also had a teratogenic effect, which corroborates the previously reported alterations for embryo-fetal development. External malformations did not differ between groups. However, the group treated with the lowest dose (EEPg 100 mg/kg) had a total frequency of malformations higher than that observed for the control group and the other doses. This fact is important and requires attention. However, a dose–response relationship was expected; that is, as the dose of EEPg increased, the frequency of malformations should increase until a plateau was reached where all fetuses were malformed or died and would be computed as post-implantation losses. However, this was not verified; that is, even with an increase in the dose of EEPg, there was no increase in malformations or the rates of post-implantation losses. According to the present design, this fact cannot be fully elucidated. However, a fact that drew attention is that the highest frequency of malformations is of the mild hydrocephaly type. This type of malformation can regress at birth and can be detected in the offspring of mothers whose pregnancies were terminated early. Thus, prematurely collected fetuses may present these variants of normality [17,18,38]. We collected fetuses on the 18th gestational day, which, despite being early given that the pregnancy can last up to the 21st gestational day, is the day recommended by the specialized literature [17,18,38]).

Regarding skeletal malformations, it was observed that, in general, 47% of fetuses in the control group had one or more malformations. In general, these can be considered variants of normality, in particular, because the pregnancy was terminated early, and it is known that the last days and hours of pregnancy are crucial for the advancement of the ossification process. In general, these variants of normality are reduced ossifications, especially of the phalanges [25,39]. However, in the present experiment, 100% of the offspring treated with EEPg showed a delay in the ossification process, and this fact is very important and indicates a teratogenic effect, including the agenesis of bones such as interparietal, orbitotransfernoid, sternebria, xiphoid process and phalanges. Except for the agenesis of the phalanges, the other agenesis is not described as variants of normality.

## 4. Materials and Methods

### 4.1. Piper Glabratum Extract Plant Material

Leaves of *Piper glabratum* Kunth were collected in February 2017 in Dourados, Mato Grosso do Sul (latitude 2.209′37.7″ south and longitude 54055′03.2″ west). The collected plant material with flowers was prepared as an herbarium followed by standard methods. After that, we submitted to the identification process based on the characteristics features of the plant: shrub, erect stem, petiole with sheath basal leaves, smooth surface, acuminate apex and eucamptodromous venation. The inflorescence spike-type flower formed as bracts and was floral-rounded and fringed the lower portion (https://floradobrasil.jbrj.gov.br/FB12782). The species were identified by Prof. Elsie Frank-lin Guimarães from the Botanical Garden of Rio de Janeiro, RJ, Brazil, and deposited (DDMS 4412) in the herbarium of the Federal University of Grande Dourados (UFGD), MS, Brazil. The research activity was registered in the National Genetic Heritage Management System—SisGen (no. AD5B086 and no. A3C3205). The ethanolic extract of *P. glabratum* was prepared according to the instructions by Leitão et al. [4]. Even the extract tested in this research is the same as that tested by Leitão et al. [4] and therefore has the same constitution.

### 4.2. Animals

Sixty Swiss mice (*Mus musculus*) of both sexes at reproductive age were used, 40 females with an average weight of 35 g and 20 males with an average weight of 36 g. The animals were obtained from the UFMS Central Animal Facility. All animals underwent a 7-day adaptation period and were placed in mini-isolators (Alesco^®^ ventilated rack, São Paulo, SP, Brazil), lined with Pinus sp. Males were kept in isolation, and females were kept in pairs using a mini-isolator. The animals were fed standard commercial feed (Nuvital^®^, Curitiba, PR, Brazil) and filtered water under a free access system. The light was controlled by photoperiod (12 h light/12 h dark). Temperature was maintained at 22 ± 2 °C, and humidity was maintained at 55 ± 10.

The research was carried out in accordance with the protocols of the Universal Declaration of Animal Rights and with the approval of the Commission for Ethics in the Use of Animals (CEUA) of the Federal University of Mato Grosso do Sul (UFMS), under the protocol number 965/2018.

### 4.3. Experimental Design

The animals were mated overnight at a ratio of 2 females: 1 male. Pregnancy detection was performed by observing the vaginal plug, which is considered day zero of pregnancy [7,17,18,24,25,38]. The pregnant animals were randomly distributed among the experimental groups (*n* = 10): Control—the animals received the EEPg vehicle (Tween 80–1%) in the proportion of 0.1 mL/10 g of body weight (b.w.), orally (p.o.), from the 1st to the 18th gestational day (g.d.); EEPg 100, 1000 and 2000 mg/kg group—the animals received EEPg at doses of 100, 1000 and 2000 mg/kg (p.o/b.w), from the 1st to the 18th g.d., respectively. The choice of the 100 mg/kg dose was based on the effective dose described by Leitão et al. [4]. Based on the effective dose, a 10 times higher dose (1000 mg/kg) and a 20 times higher dose (2000 mg/kg) were defined, following the recommendations of the area’s guidelines [40,41,42].

### 4.4. Biological Tests

On the 18th g.d. the animals were submitted to euthanasia followed by laparotomy, hysterectomy, omphalectomy and thoracotomy for collection, weighing and proper storage of organs (lung, heart, spleen, liver, kidneys, placenta) and fetuses.

The collected fetuses were weighed and underwent systematic analysis of external malformations and sexing. They were then randomly assigned to two subgroups, each comprising approximately 50% of the litter. Fetuses from the first subgroup were fixed in absolute acetone for at least seven days and destined for skeletal analysis using the technique described by Staples and Schnell [43] and modified by Oliveira et al. [7]. After fixation, the fetuses were eviscerated and immersed in a KOH solution (0.8%) for the diaphanization process. Then, four drops of Alizarin Red were added. This solution was changed every 24 h for four consecutive days. After staining the fetuses, the KOH solution was replaced by the bleaching solution (1 L of glycerin: 1 L of ethyl alcohol: 0.5 L of benzyl alcohol) and changed every 24 h for five consecutive days. Malformations were classified based on studies by Taylor [1], Manson et al. [44], Wise et al. [45], Damasceno et al. [46] and Oliveira et al. [7]. Fetuses from the second subgroup were fixed in Bodian’s solution (distilled water (142 mL), acetic acid (50 mL), formaldehyde (50 mL) and 95% alcohol (758 mL)) for at least seven days and sent for analysis visceral [19] through microdissection with strategic cuts, for the study of the thorax and abdomen, proposed by Barrow and Taylor [47], and for the study of the head, according to Wilson [48], with alterations proposed by Oliveira et al. [49], Damasceno et al. [46] and Oliveira et al. [7]. The classification of visceral and skeletal alterations was the basis of the work of Taylor [50], Manson and Kang [51], Wise et al. [45], Damasceno et al. [46] and Oliveira et al. [7]. Analyzes of viscera and fetal skeletons were performed using a stereomicroscope (Nikon^®^—SMZ 745T, New York, NY, USA) with a 4×magnification.

### 4.5. Biometric Parameters

The biometric parameters were calculated according to data on initial weight (weighed females on day zero), final weight (weighed females on the 18th g.d.), weight gain (final weight–initial weight), uterine weight and net weight gain (weight gain–uterus weight), in addition to data on absolute and relative weights (organ weight / final weight) of the heart, lung, spleen, kidneys and liver.

### 4.6. Reproductive Performance and Embryofetal Development

The reproductive parameters related to the number of implants (number of live fetuses + number of dead fetuses + number of resorptions) and number of live fetuses were quantified and/or calculated; fetal viability (no. of live fetuses *100/no. of implants), post-implantation loss rate ((no. of implants—no. of live fetuses) *100/no. of implants), number of resorptions, resorption rate (no. of resorptions *100/number of implants), placental weight, fetal weight, placental index (placental weight/fetal weight), placental efficiency (fetal weight / placental weight) and sex ratio (number of male fetuses *100/number of female fetuses) were also quantified or calculated.

Classification of fetal weight according to gestational age (CFWGA) was carried out in two ways: (I) according to Soulimane-Mokhtari et al. [23], fetuses were considered suitable for gestational age (SUGA) when they did not differ more than ± 1.7× standard deviation (SD) from the mean of control fetuses, were considered small for gestational age (SGA) when they present body weight less than 1.7× standard deviation in relation to the mean of fetuses in the control group and were considered large for gestational age (LGA) when they present body weight greater than + 1.7× standard deviation in relation to the mean of the control group, and (II) according to Oliveira et al. [7], fetuses were classified as fetuses with appropriate weight for age at pregnancy (AWAP) when the weight of the fetus was between the mean weight of the fetuses in the control group plus or minus the standard deviation, were classified with low weight for the age of pregnancy (LWAP) when the weight of the fetus is lower than the mean weight of fetuses in the control group minus the standard deviation of this same group and were classified with high weight for the age of pregnancy (HWAP) when the weight of the fetus is greater than the mean weight of fetuses in the control group plus the standard deviation of this same group. Soulimane-Mokhtari et al. [23] classify the weights of fetuses individually, and Oliveira et al. [7] classify the litter in general.

### 4.7. Statistical Analysis

The ANOVA/Tukey test and the Chi-square test were used to compare frequencies between groups. Data were presented as mean ± standard error of mean or mean ± standard deviation, and the established significance level was *p* < 0.05.

## 5. Conclusions

In view of the above, it is considered that EEPg has a low maternal toxic potential and does not alter reproductive performance. However, it has teratogenic potential. The teratogenic effect was evidenced mainly by the interference in the ossification process. Therefore, EEPg is contraindicated for use during pregnancy.

## Figures and Tables

**Table 1:** Mean values ± standard error of the mean of the biometric parameters of females treated with the ethanolic extract of *P. glabratum* (EEPg).

Biometric Parameters (g)					
Experimental Groups	Initial Weight	Final Weight	Weight Gain	Uterus Weight	Net Weight Gain
Control	36.56 ± 1.31 ^a^	53.72 ± 1.47 ^a,b^	17.16 ± 1.93 ^a,b^	17.65 ± 1.28 ^a,b^	−0.50 ± 1.31 ^a^
EEPg 100 mg/kg	35.42 ± 2.00 ^a^	55.97 ± 1.42 ^b^	20.55 ± 1.27 ^b^	18.95 ± 0.48 ^b^	1.60 ± 1.10 ^a^
EEPg 1000 mg/kg	34.51 ± 1.00 ^a^	48.62 ± 1.75 ^a^	14.28 ± 2.03 ^a^	14.40 ± 1.49 ^a^	−0.11 ± 0.98 ^a^
EEPg 2000 mg/kg	35.04 ± 2.01 ^a^	47.73 ± 1.92 ^a^	12.69 ± 1.11 ^a^	14.11 ± 1.03 ^a^	−1.42 ± 0.78 ^a^

g—grams. EEPg—ethanolic extract of *Piper glabratrum*. Different letters indicate statistically significant differences. Mean + Standard error of the mean (Statistical Test: Anova/Tukey; *p* < 0.05).

**Table 2:** Mean values ± standard error of mean absolute and relative weights of the organs of females treated with the ethanolic extract of *P. glabratum* (EEPg).

The absolute weight of organs (g)					
Experimental Groups	Heart	Lungs	Liver	Kidney	Spleen
Control	0.20 ± 0.01 ^a^	0.25 ± 0.01 ^a^	2.44 ± 0.11 ^a^	0.44 ± 0.02 ^a^	0.19 ± 0.02 ^b^
EEPg 100 mg/kg	0.18 ± 0.01 ^a^	0.23 ± 0.01 ^a^	2.45 ± 0.04 ^a^	0.42 ± 0.02 ^a^	0.17 ± 0.02 ^a,b^
EEPg 1000 mg/kg	0.18 ± 0.01 ^a^	0.27 ± 0.01 ^a^	2.37 ± 0.06 ^a^	0.41 ± 0.03 ^a^	0.13 ± 0.01 ^a,b^
EEPg 2000 kg/kg	0.17 ± 0.01 ^a^	0.26 ± 0.01 ^a^	2.62 ± 0.07 ^a^	0.39 ± 0.02 ^a^	0.12 ± 0.01 ^a^
Relative weight of organs					
Experimental Groups	Heart	Lungs	Liver	Kidney	Spleen
Control	0.0037 ± 0.0002 ^a^	0.0047 ± 0.0001 ^a^	0.045 ± 0.0018 ^a^	0.0081 ± 0.0003 ^a^	0.0036 ± 0.0004 ^a^
EEPg 100 mg/kg	0.0032 ± 0.0001 ^a^	0.0041 ± 0.0002 ^a^	0.044 ± 0.0009 ^a^	0.0075 ± 0.0002 ^a^	0.0030 ± 0.0004 ^a^
EEPg 1000 mg/kg	0.0038 ± 0.0003 ^a^	0.0056 ± 0.0003 ^b^	0.049 ± 0.0015 ^a^	0.0085 ± 0.0006 ^a^	0.0027 ± 0.0002 ^a^
EEPg 2000 mg/kg	0.0035 ± 0.0001 ^a^	0.0054 ± 0.0002 ^b^	0.055 ± 0.0019 ^b^	0.0083 ± 0.0003 ^a^	0.0025 ± 0.0002 ^a^

g—grams. EEPg—ethanolic extract of *Piper glabratrum*. Different letters indicate statistically significant differences. Mean + Standard error of the mean (Statistical Test: Anova/Tukey; *p* < 0.05).

**Table 3:** Mean values ± standard error of mean parameters related to reproductive performance of females treated with ethanolic extract of *P. glabratum* (EEPg).

Parameters	Experimental Groups			
	Control	EEPg 100 mg/kg	EEPg 1000 mg/kg	EEPg 2000 mg/kg
Number of pregnant females	10	10	10	10
Implants	13.50 ± 1.07 ^a^	14.40 ± 0.34 ^a^	12.89 ± 1.41 ^a^	13.10 ± 1.08 ^a^
Live fetuses	12.10 ± 0.90 ^a^	13.20 ± 0.44 ^a^	11.22 ± 1.36 ^a^	11.10 ± 0.85 ^a^
Dead fetuses	0.10 ± 0.10 ^a^	0.30 ± 0.15 ^a^	0.11 ± 0.11 ^a^	0.10 ± 0.10 ^a^
Fetal Viability	90.85 ± 3.28 ^a^	91.80 ± 2.69 ^a^	88.40 ± 4.97 ^a^	85.62 ± 2.96 ^a^
Resorption	1.30 ± 0.52 ^a^	1.00 ± 0.37 ^a^	1.56 ± 0.73 ^a^	1.90 ± 0.53 ^a^
RPIL	9.15 ± 3.28 ^a^	8.20 ± 2.69 ^a^	11.60 ± 4.97 ^a^	14.38 ± 2.96 ^a^
RR	8.56 ± 3.28 ^a^	6.82 ± 2.50 ^a^	10.86 ± 4.87 ^a^	13.79 ± 3.08 ^a^
Sex ratio	1.43 ± 0.16 ^a^	2.68 ± 1.03 ^a^	2.32 ± 1.00 ^a^	1.11 ± 0.23 ^a^

RPIL—Rate of post-implantation losses; RR—Reabsorption rate. EEPg—ethanolic extract of *Piper glabratrum*. Equal letters indicate an absence of statistically significant differences. Mean + Standard error of the mean (Statistical Test: Anova/Tukey; *p* > 0.05).

**Table 4:** Mean values ± standard error of mean parameters related to embryofetal development of females treated with ethanolic extract of *P. glabratum* (EEPg).

Parameters	Experimental Groups			
	Control	EEPg 100 mg/kg	EEPg 1000 mg/kg	EEPg 2000 mg/kg
Fetal weight *	1.12 ± 0.11 ^b^	1.13 ± 0.12 ^b^	1.02 ± 0.17 ^a^	1.02 ± 0.01 ^a^
Placenta weight	0.075 ± 0.002 ^b^	0.075 ± 0.001 ^b^	0.059 ± 0.002 ^a^	0.075 ± 0.001 ^b^
Placental Index	0.067 ± 0.001 ^b^	0.067 ± 0.001 ^b^	0.059 ± 0.002 ^a^	0.076 ± 0.002 ^c^
Placental Efficiency	15.77 ± 0.35 ^a^	15.61 ± 0.27 ^a^	19.32 ± 0.73 ^b^	14.23 ± 0.40 ^a^
SGA	3.31%	6.11%	32.67% *	24.32% *
SUGA	92.56%	91.60%	62.38%	74.77%
LGA	4.13%	2.29%	4.95%	0.90%
AWPA	-	AWPA	AWPA	AWPA

CFWGA—classification of fetal weight according to gestational age; SGA—small for gestational age; SUGA—suitable for gestational Age; LGA—large for gestational age. AWPA [7]—adequacy of weight to pregnancy age. EEPg—ethanolic extract of *Piper glabratrum*. Different letters indicate statistically significant differences. *Significant difference compared to the control group. Mean + Standard error of the mean (Statistical Test: Anova/Tukey; *p* < 0.05).

**Table 5:** Absolute number and percentage of external malformations found in the offspring of females treated with the ethanolic extract of *P. glabratum* (EEPg).

Parameters	Experimental Groups			
	Control	EEPg 100 mg/kg	EEPg 1000 mg/kg	EEPg 2000 mg/kg
External Analysis				
Analyzed fetuses	121	131	101	111
Normal fetuses	106	109	94	100
%	87.60	83.21	93.07	90.10
Hydropsy	0	0	0	1
%	0.00	0.00	0.00	0.90
Kyphosis	1	10 *	1	4
%	0.83	7.63	0.99	3.60
Unilateral forelimb hyperflexion	1	3	1	0
%	0.83	2.29	0.99	0.00
Unilateral hindlimb hyperflexion	0	1	3	1
%	0	0.76	2.97	0.90
Bilateral hind limb hyperflexion	0	0	1	1
%	0.00	0.00	0.99	0.90
Unilateral forelimb hyperextension	2	1	1	0
%	1.65	0.76	0.99	0.00
Unilateral hind limb hyperextension	7	2	0	0
%	5.79	1.53	0.00	0.00
Bilateral hind limb hyperextension	0	1	0	0
%	0.00	0.76	0.00	0.00
Small paw	0	2	0	1
%	0.00	1.53	0.00	0.90
Bent tail	4	2	0	2
%	3.31	1.53	0.00	1.80
Frequency of external malformation	15	22	12	11
% external malformation	12.40	16.79	6.93	9.90

%—Percentage of fetuses with malformations. EEPg—ethanolic extract of *Piper glabratrum*. * Statistically significant difference compared to Control. Absolute numbers (Statistical Test: Chi-square; *p* < 0.05).

**Table 6:** Absolute number and percentage of visceral malformations found in the offspring of females treated with the ethanolic extract of *P. glabratum* (EEPg).

Parameters	Experimental Groups			
	Control	EEPg 100 mg/kg	EEPg 1000 mg/kg	EEPg 2000 mg/kg
Visceral Analysis				
Analyzed fetuses	53	62	53	45
Normal fetuses	43	34	39	30
%	81.13	54.84	73.58	66.67
Severe hydrocephaly	1	2	2	1
%	1.89	3.23	3.77	2.22
Moderate hydrocephaly	0	3	3	6 *
%	0.00	4.84	5.66	13.33
Mild hydrocephaly	9	18	8	8
%	16.98	29.03	15.09	17.78
Choanae hyperplasia	0	1	0	0
%	0.00	1.61	0.00	0.00
Forked tongue	0	2	0	0
%	0.00	3.23	0.00	0.00
Enlarged ventricular chamber	0	2	1	0
%	0.00	3.23	1.89	0.00
Visceral malformation frequency	10	28 *	14	15
% visceral malformation	18.87	45.16	16.42	33.33

Percentage of fetuses with malformations. EEPg—ethanolic extract of *Piper glabratrum*. * Statistically significant difference compared to Control. Absolute numbers (Statistical Test: Chi-square; *p* < 0.05).

**Table 7:** Absolute number and percentage of skeletal malformations found in the offspring of females treated with the ethanolic extract of *P. glabratum* (EEPg).

Parameters	Experimental Groups			
	Control	EEPg 100 mg/kg	EEPg 1000 mg/kg	EEPg 2000 mg/kg
Skeletal Analysis				
Analyzed fetuses	57	67	51	57
Normal fetuses	30	0	0	0
%	52.63	0.00	0.00	0.00
Head				
Interparietal agenesis	1	0	0	0
%	1.75	0.00	0.00	0.00
Right orbitotransphenoid agenesis	1	0	0	0
%	1.75	0.00	0.00	0.00
RO Frontal	0	16 *	40 *	57 *
%	0.00	23.88	78.43	100.00
RO Interparietal	3	43 *	31 *	46 *
%	5.26	64.18	60.78	80.7
RO Maxilla	0	2	0	0
%	0.00	2.99	0.00	0.00
RO Nasal	2	43 *	50 *	57 *
%	3.51	64.18	98.04	100.00
RO Parietal	2	43 *	48 *	57 *
%	3.51	64.18	94.12	100.00
RO premaxilla	0	2	2	0
%	0.00	2.99	3.92	0.00
RO supraoccipital	0	15 *	7 *	10 *
%	0.00	22.39	13.73	17.54
RO zygomatic	0	2	0	0
%	0.00	2.99	0.00	0.00
Trunk				
Agenesis of sternebral center	0	1	6 *	5
%	0.00	1.49	11.76	8.77
Agenesis of the Xiphoid Process	1	0	0	0
%	1.75	0.00	0.00	0.00
Arthrogryposis	0	0	0	1
%	0.00	0.00	0.00	1.75
Misshapen sternebra	9	34 *	20 *	21 *
%	15.79	50.75	39.22	36.84
Forked rib	0	0	0	1
%	0.00	0.00	0.00	1.75
Hyperkyphosis	0	1	0	0
%	0.00	1.49	0.00	0.00
Hyperkyphosis with head forward	0	1	0	0
%	0.00	1.49	0.00	0.00
RO Rib	0	0	0	2
%	0.00	0.00	0.00	3.51
RO sternebra	6	12	19 *	16 *
%	10.53	17.91	37.25	28.07
RO Xiphoid Process	0	2	3	3
%	0.00	2.99	5.88	5.26
Rotation of the vertebral bodies	0	1	0	5
%	0.00	1.49	0.00	8.77
Members				
Phalanx agenesis	1	2	17 *	15 *
%	1.75	2.99	33.33	26.32
Agenesis of the metacarpal	1	0	0	0
%	1.75	0.00	0.00	0.00
Agenesis of the caudal arch	0	0	0	2
%	0.00	0.00	0.00	3.51
Alteration of the nucleus of ossification of the knee and elbow	0	0	0	2
%	0.00	0.00	0.00	3.51
Frequency of skeletal malformation	27	67 *	51 *	57 *
% skeletal malformation	47.37	100	100	100

%—Percentage of fetuses with malformations; RO—Reduced Ossification. EEPg—ethanolic extract of *Piper glabratrum*. * Statistically significant difference compared to Control. Absolute numbers (Statistical Test: Chi-square; *p* < 0.05).

## Data Availability

Data is contained within the article.

## Abbreviations

AWPA	adequacy of weight to pregnancy age;
b.w	body weight
CEUA	commission for Ethics in the Use of Animals
CFWGA	classification of fetal weight to gestational age;
EEPg	ethanolic extract of *Piper glabratrum*
g	grams.
g.d	gestational day
HWAP	high weight for the age of pregnancy
LD_50_	lethal dose 50
LGA	large for gestational age.
LWAP	low weight for the age of pregnancy
RO	reduced Ossification
RPIL	rate of post-implantation losses;
RR	reabsorption rate
SD	standard deviation
SGA	small for gestational age
SUGA	suitable for gestational Age;
UFMS	Federal University of Mato Grosso do Sul
Unicef	United Nations Children’s Fund
